# Synoptic Reporting Improves Quality of Endobronchial Ultrasound (EBUS): An Australian Multicentre Study

**DOI:** 10.3390/cancers18101528

**Published:** 2026-05-09

**Authors:** Ashleigh Witt, Nicole M. Rankin, Hanson Siu, Nicholas Wilsmore, Shaun Yo, Christopher Lyne, Kexin Sun, Kanishka Rangamuwa, Tracy Leong, Ashleigh Hocking, Shankar Siva, Daniel P. Steinfort

**Affiliations:** 1 Department of Medicine, Faculty of Medicine, Dentistry & Health Sciences, University of Melbourne, Parkville, Melbourne, VIC 3050, Australia; 2 Department of Respiratory Medicine, Royal Melbourne Hospital, Parkville, Melbourne, VIC 3052, Australia; 3 Melbourne School of Population and Global Health, University of Melbourne, Parkville, Melbourne, VIC 3050, Australia; 4 Department of Respiratory Medicine, Eastern Health, Box Hill, Melbourne, VIC 3128, Australia; 5 Department of Respiratory Medicine, Monash Health, Clayton, Melbourne, VIC 3168, Australia; 6 Department of Respiratory Medicine, Northern Health, Epping, Melbourne, VIC 3076, Australia; 7 Department of Respiratory Medicine, Austin Health, Heidelberg, Melbourne, VIC 3084, Australia; tracy.leong@austin.org.au (T.L.);; 8 Peter MacCallum Cancer Centre, Parkville, Melbourne, VIC 3052, Australia

**Keywords:** lung cancer, staging, synoptic reporting, endobronchial ultrasound

## Abstract

**Introduction**: Accurate mediastinal staging with endobronchial ultrasound (EBUS) is essential in determining prognosis and management of patients with non-small cell lung carcinoma (NSCLC). Procedural documentation is often variable and incomplete due to reliance on free-text reporting. Synoptic reporting, which is a structured, standardised approach, improves documentation quality in other specialties, but has not been evaluated in EBUS. This study aimed to evaluate the impact of synoptic reporting on quality metrics in systematic staging EBUS. **Methods**: This multicentre ambispective study was conducted across five Australian tertiary hospitals. Synoptic reporting was implemented for EBUS procedures where systematic staging is recommended. Outcomes from three months of pre-intervention data were compared with three months post-intervention. The primary outcome was completeness of systematic mediastinal staging. Secondary outcomes included number of lymph nodes sampled per procedure, number of radiologically normal nodes sampled, detection of positron emission tomography (PET)-occult lymph node metastases, and report completeness. **Results**: Ninety-six procedures were included pre-intervention, and 77 post-intervention. Complete mediastinal staging increased from 17/96 (17.7%) to 67/77 (87%) (OR 3.2; *p* < 0.001). The mean number of lymph nodes sampled per procedure increased from 1.75 to 2.45 (*p* < 0.001), and sampling of radiologically normal nodes increased from 37/96 (38.5%) to 55/77 (71.4%) (OR 3.95; *p* < 0.001). Detection of PET-occult nodal metastases increased from 3/96 (3.1%) to 9/77 (11.7%) (OR 4.07; *p* = 0.036). Report completeness improved across most mandated domains. **Conclusions**: Synoptic procedural improves the quality and performance of systematic staging EBUS and represents a scalable quality improvement intervention.

## Simple Summary

Synoptic reporting is a structured way of documenting medical procedures using standardised fields rather than free-text. In endobronchial ultrasound (EBUS), a procedure used to diagnose and stage lung cancer, reporting quality can vary and important details may be missed. In this study, we evaluated whether synoptic reporting improved the quality and performance of EBUS procedures across five Australian hospitals. Following implementation of synoptic reporting, rates of complete staging procedures increased significantly, more lymph nodes were biopsied, and detection of previously unrecognised cancer spread improved. Synoptic reporting is a simple and effective way to improve the quality of lung cancer staging procedures.

## 1. Introduction

Accurate staging of the mediastinum is critical for prognostication and treatment selection in non-small cell lung carcinoma (NSCLC) [1,2,3], as nodal status (N0 through to N3) remains one of the most important determinants of therapeutic strategy [4,5]. Patients with N0-N1 disease are typically managed with curative-intent surgical resection, often with consideration of adjuvant systemic therapy [6,7]. In contrast, the presence of N2-N3 disease generally shifts management towards multimodality or non-surgical approaches such as chemoradiotherapy and immunotherapy [8]. This reflects more advanced disease biology and reduced likelihood of cure with surgical resection alone. Failure to identify N2 disease may lead to inappropriate surgical resection, when combined modality treatment may be more appropriate [9]. Conversely, accurate exclusion of mediastinal disease supports appropriate selection for curative intent surgery. Endobronchial ultrasound (EBUS) is the preferred method of mediastinal staging due to its accuracy and safety [2].

The World Association of Bronchology and Interventional Pulmonology (WABIP) recently proposed quality indicators and standardised reporting elements for EBUS [10]. However, there is no established format for EBUS procedural reporting [10]. As a result, the quality and completeness of EBUS procedural documentation remains highly variable, often relying on narrative or free-text formats [11]. Free-text reporting may result in omission of key procedural elements, limiting the ability to assess adherence to quality guidelines [10], and potentially impacting patient outcomes.

In contrast to narrative free-text reports, synoptic reporting uses structured, checklist-based templates with mandatory data fields to ensure consistent documentation of all clinically relevant information [12,13]. Synoptic reporting has been shown to improve report completeness, facilitate audit, and improve both the accuracy and reliability of oncological data across multiple specialties including surgery and gastrointestinal endoscopy [13,14,15,16], as well as pathology and radiology [17,18]. In lung cancer specifically, deficiencies in pathology reporting have been well described, with omission of critical elements such as tumour stage and nodal status impacting prognostication and treatment decisions [19]. In lung cancer pathology, synoptic reporting combined with education interventions significantly increased the likelihood of complete reporting of essential items and improved accuracy of pT and pN staging [19].

Systematic mediastinal staging EBUS is the sequential assessment of the five key mediastinal stations (station 2R, 2L, 4R, 4L, 7) and is recommended in numerous international clinical practice guidelines [10,20]. We previously demonstrated the impact of inadequate procedural reporting on the ability to establish quality metrics in EBUS [11]. To date, no published studies have evaluated synoptic procedural reporting in interventional pulmonology, including systematic staging EBUS. This multicentre Australian study aimed to investigate the effect of implementing a synoptic procedural report for systematic staging EBUS across five Australian tertiary hospitals, and to establish the impact on quality indicators. We hypothesised that the implementation of synoptic reporting would improve completeness and quality of systematic mediastinal staging and result in improved detection of clinically relevant nodal disease.

## 2. Methods

### 2.1. Study Design

This multicentre implementation study across five Australian tertiary hospitals used an ambispective design, with retrospective and prospective reports collected during three-month periods before and after implementation of synoptic reporting. Synoptic reporting was implemented on 1 November 2024, with pre- and post-intervention study periods spanning three months either side of this.

This study was approved by the Melbourne Health Human Research Ethics Committee (HREC/113533/MH-2024.364). A waiver of consent was granted as the study involved no change to routine clinical care and posed minimal risk to participants.

### 2.2. Hospital and Patient Selection

Participating centres were metropolitan hospitals in Melbourne, Australia, with established lung cancer services that routinely perform systematic mediastinal staging EBUS for patients with clinical stage IB-IIIC NSCLC.

Eligible patients were consecutive adults (aged ≥18 years) undergoing systematic staging EBUS for suspected or confirmed clinical stage IB-III NSCLC during a 3-month pre-intervention period (retrospective cohort) and 3-month post-intervention period (prospective cohort).

### 2.3. Study Procedure

The retrospective phase involved examining historical narrative style reports generated in routine clinical practice in the time period leading to the intervention start date. Additional data sources examined in both phases were pathology reports from the EBUS procedure and Positron Emission Tomography (PET) and/or Computed Tomography (CT) reports for each patient. For patients who proceeded to surgical resection following their EBUS procedure, surgical pathology reports were reviewed to analyse rate of surgical upstaging.

Radiologically normal nodes were defined as lymph nodes without enlargement on CT or FDG avidity on PET. Morphologically suspicious nodes were either enlarged or demonstrated FDG avidity. PET-occult metastases were defined as histologically confirmed metastases in lymph nodes without enlargement or FDG uptake on PET/CT. No fixed FDG SUVmax threshold was applied, with PET interpretation based on routine clinical reporting by centres.

Sites submitted all EBUS cases performed for any indication during the study period to allow analysis of whether patients eligible for staging had a staging procedure. In cases where reports did not specify staging, procedures were deemed to be staging if reports described evaluation (EBUS image appearance and/or sampling) of >2 lymph nodes in a sequential fashion [11].

During the post-intervention phase, proceduralists used a synoptic reporting template in existing reporting software (Endobase Version III, Olympus Australia or Provation MD Version 5.0, Provation Medical Australia) with mandated fields. Table 1 outlines the mandated fields.

Across participating sites, procedures were performed by experienced proceduralists, with multiple clinicians per site as follows: Site 1 (*n* = 2), Site 2 (*n* = 5), Site 3 (*n* = 2), Site 4 (*n* = 4), Site 5 (*n* = 2).

Three sites implemented synoptic reporting using templates integrated within the Endobase reporting software. Key data fields were embedded as structured, mandatory elements that required completion prior to report finalisation. Drop down menus were triggered when ‘systematic staging EBUS’ was selected as the indication for the procedure. In particular, it was mandated that for each mediastinal lymph node (2R, 2L, 4R, 4L, 7), comment should be made on whether it was examined, its size and the number of needle passes.

At the two remaining sites, synoptic reporting was implemented in a non-integrated format. Proceduralists were provided with the same predefined reporting framework and mandated data element; however, these fields were not embedded in the reporting software as structured fields. Instead, clinicians entered the required elements within the narrative free-text report, without system-level enforcement.

### 2.4. Study Objectives and Outcomes

The objective of this study was to evaluate the impact of synoptic procedural reporting on the quality and performance of systematic staging EBUS across multiple centres. The primary outcome was the proportion of patients undergoing complete systematic mediastinal staging, defined as an examination of all five mediastinal lymph node stations, with sampling of nodes ≥5 mm or that were morphologically suspicious.

Secondary outcomes included rate of detection of PET-occult lymph node metastases, number of lymph nodes sampled, proportion of patients with at least one radiologically normal node sampled, and report completeness, defined as fidelity to the five mandated reporting items. For patients who underwent surgical resection, rate of surgical upstaging was recorded.

### 2.5. Statistical Analyses

The primary outcome was compared between pre- and post-intervention cohorts using Fishers exact test. The mean numbers of lymph nodes sampled were compared using a Welch two-sample *t*-test. Comparative analyses were performed to evaluate differences in outcomes between integrated and non-integrated synoptic reporting approaches, with interaction testing used to assess whether the effect of the intervention varied by method of implementation.

A generalised linear mixed-effects model with a binomial distribution and logit link was used to evaluate the association between the intervention and complete systematic mediastinal staging, with study site included as a random intercept to account for clustering.

A formal sample size calculation was not performed. This study was designed to include all consecutive eligible cases within predefined study periods, and analyses should therefore be considered exploratory.

Statistical analyses were performed using R (version 2026.04.0+526; R Foundation for Statistical Computing, Vienna, Austria).

## 3. Results

### 3.1. Patient Characteristics

Across the five sites, a total of 395 linear EBUS procedures were performed, of which 173 were systematic staging EBUS procedures. Supplementary Figure S1 shows a breakdown of all systematic staging EBUS procedures, identifying staging procedures amongst other indications. Baseline demographics of included patients are represented in Table 2. The median age was 70 years (IQR 61–76), and 68 (39%) patients were female.

### 3.2. Primary Outcome

Complete systematic mediastinal staging was observed in 17/96 (17.7%) of the historical cohort, compared with 67/77 (87%) in the synoptic cohort (odds ratio (OR) 3.2, 95% CI 1.38–7.5; *p* < 0.001. A high degree of variance in care was noted pre-intervention, with the proportion of complete systematic mediastinal staging varying from 3.1% to 45.5%. Table 3 delineates completeness of staging by study site, demonstrating improvements across all study sites, four of which were statistically significant. Prior to intervention, Site 5 had substantially higher rates of complete staging procedures improving to 100% completeness rate with the intervention. Variance in care between sites was markedly reduced following introduction of synoptic reporting.

### 3.3. Secondary Outcomes

#### 3.3.1. Nodal Metastasis Detection

Detection of PET-occult lymph nodes was observed in 3/96 (3.1%) pre-intervention, increasing to 9/77 (11.7%) post-intervention (Fisher’s exact text, OR 4.07, 95% CI 0.97–24.24; *p* = 0.036).

In pre-intervention cohort, pathological upstaging occurred in 3 of 96 cases (3.1%). Following implementation of synoptic reporting, the rate of upstaging increased to 9 of 77 cases (11.7%). This represents a statistically significant upstaging between the pre- and post-intervention periods (Fisher’s exact test, *p* = 0.036).

#### 3.3.2. Number of Nodes Sampled

The mean number of lymph nodes sampled increased from 1.75 (SD 1.12) pre-intervention to 2.45 (SD 1.32) post-intervention (mean difference 0.70; Welch two-sample *t*-test, *p* < 0.001). The median number of nodes sampled remained 2.0 in both cohorts.

The proportion of patients with at least one radiologically normal lymph node sampled increased from 37/96 (38.5%) pre-intervention to 55/77 (71.4%) post-intervention (Fisher’s exact test, OR 3.95, 95% CI 2.00–8.00; *p* < 0.001).

#### 3.3.3. Post Surgical Upstaging

Of patients who proceeded to surgical resection following their staging EBUS, 2/23 (8.7%) were upstaged post-operatively in the pre-intervention cohort, compared to 1/28 (3.6%) in the post-intervention cohort (*p* = 0.58). This is broken into sites in Table 4.

#### 3.3.4. Report Completeness

As shown in Table 5, fidelity to the five mandated reporting items increased significantly across four of the six domains following implementation of synoptic reporting. The proceduralist’s name was well documented in both cohorts and showed minimal change, and documentation of immediate adverse events was unchanged. The proportion of procedures containing all six reporting domains increased from 3.1% (3/96) to 16.9% (13/77) (OR 6.23, CI 1.62–35.46; *p* = 0.003).

### 3.4. Comparison in Synoptic Reporting Techniques

Comparison of the two methods of synoptic reporting (integrated and non-integrated) are shown in Table 6. All outcomes showed positive improvements but were not statistically significant. Sites with non-integrated reporting showed larger improvements consistent with greater scope for improvement.

### 3.5. Site Variability

Implementation of the synoptic reporting intervention was associated with a marked increase in the odds of complete systematic mediastinal staging procedure (β = 4.02, SE 0.54; *p* < 0.001). This corresponds to an approximate 56-fold increase in odds. There was moderate between-site variability (random intercept variance 0.81, SD 0.90). Overall, the effect of the intervention remained highly significant after accounting for clustering by site (χ^2^ = 55.16, *p* < 0.001).

## 4. Discussion

This study aimed to evaluate the impact of synoptic procedural reporting on the quality and performance of systematic staging EBUS. Implementation of synoptic reporting was associated with marked improvements in complete systematic mediastinal staging and several key procedural quality metrics. It is the first study to demonstrate the utility and advantage of synoptic reporting in systematic staging EBUS, and interventional pulmonology more broadly. These findings indicate that synoptic reporting may play an important role in reducing variance in care for systematic staging EBUS procedures, and in improving quality metrics for both process and outcome measures in EBUS.

The primary outcome of complete systematic mediastinal staging increased significantly following the implementation of synoptic reporting. By prompting the proceduralist to systematically consider each mediastinal lymph node station, the structured format of synoptic reports reduces the likelihood that stations are overlooked. Requiring documentation of each nodal station encourages a more deliberate and accountable approach to the procedure. Similar effects of structured reporting prompting more thorough examination has been described in other domains of cancer care [21]. Improved mediastinal staging may influence treatment selection and long-term outcomes. Accurate nodal assessment is critical in guiding treatment decisions, particularly with the increasing use of neoadjuvant immunochemotherapy [22,23].

Although numerous studies across medical specialties have demonstrated that synoptic reporting improves the completeness and quality of clinical documentation, most have focused primarily on reporting metrics rather than clinical outcomes. In contrast, our findings suggest that the benefits of synoptic reporting extend beyond improved documentation alone. Synoptic reporting was associated with improvements in diagnostic performance, including increased detection of clinically significant PET-occult nodal disease. These findings support the role of synoptic reporting not only as a documentation tool, but as a strategy to enhance the quality and effectiveness of cancer staging procedures. This is particularly relevant given that cancer staging is frequently incompletely captured [24,25], limiting accurate benchmarking and system-level performance evaluation.

In addition to improving documentation, synoptic reporting may have a role in training and standardising practice for less experienced proceduralists. Structured templates provide real-time prompts that may support adherence to recommended staging approaches.

The magnitude of improvement varied across the study sites. At sites with lower baseline performance, synoptic reporting was associated with substantial absolute improvements. Sites 1, 3 and 4 increased from historical complete systematic mediastinal staging rates of 3.1%, 14.3% and 10.3% respectively to post-intervention rates exceeding 75% at each site. Site 2 demonstrated a numerical improvement that did not reach statistical significance, likely reflecting smaller case numbers. Site 5 had a higher historical staging completeness than other sites which improved further to 100% in the synoptic cohort.

This study demonstrates, for the first time, that synoptic reporting is associated with improvements in several key quality indicators recommended by WABIP [10]. Synoptic reporting was associated with more complete systematic staging procedures, resulting in increased diagnosis of lymph node metastases and thus changing patient management. With synoptic reporting, more lymph nodes were sampled per procedure and more radiologically normal nodes sampled. The most relevant clinical outcome measured in this study was detection of PET-occult lymph node metastases which showed a statistically significant increase 3.1% to 11.7%, which is more consistent with published meta-analyses [26].

Whilst increased detection of nodal metastases is clinically meaningful, this study did not capture downstream changes in management decisions. In practice, detection of PET-occult nodal disease would be expected to alter treatment pathways in a proportion of patients, including eligibility for surgical resection. The direct impact of synoptic reporting on treatment decisions and patient outcomes remains less clearly established. It is likely to be mediated through enhanced multidisciplinary decision-making and warrants further study.

The mechanism of implementation of synoptic reporting varied between sites and likely contributed in part to the heterogeneity in effect size between institutions. Differences in reporting templates, including the presence of mandated fields, may influence clinician behaviour through varying degrees of prompting. However, despite these differences, improvements were consistently observed across all sites, suggesting that the intention to report synoptically rather than the specific mode of implementation was the driver of change.

Whilst mandated fields may be helpful for some clinicians, our experience suggested that this did not meaningfully influence outcomes. Although interaction testing did not demonstrate a statistically significant difference between integrated and non-integrated reporting systems, there were observable differences in the magnitude of improvement across several outcomes. In particular, non-integrated systems demonstrated larger absolute improvements in complete staging procedures and PET-occult lymph node detection. This suggests synoptic reporting has potentially greater gains where reporting infrastructure is not already established.

Our findings are consistent with those reported in other specialities [12,13,27], where synoptic reporting has been shown to improve not only documentation and medical records, but also patient outcomes [28,29]. While improvements in documentation are important for audit and performance monitoring, the ultimate goal of respiratory physicians is improved cancer detection and patient survival. In this study, the implementation of synoptic reporting was associated with improvements in both the quality of procedural reports and measures of diagnostic performance.

Improvements were not uniform across all reporting domains. Documentation of clinical stage showed modest gains, likely reflecting reliance on external data, and hence were less amenable to structured reporting alone.

A key strength of this study is the inclusion of all EBUS procedures in the study period to minimise the risk of selection bias. The ambispective study design allowed the direct measurement of the impact of the study intervention, which strengthens causal inference. Improvements were demonstrated across diverse baseline performance, supporting the strength of the intervention effect.

Some limitations must be acknowledged. The pre–post design without randomisation introduces potential temporal confounding and limits causal inference. Procedural performance was not independently validated, and outcomes relied on reported data. Outcomes were analysed at the institutional level; however, procedural performance is influenced by individual clinician factors, including experience and case volume. Detailed case volume per proceduralist was not available, limiting analysis of clinician-level effects. It is possible clinician experience contributed to both baseline performance and some observed improvements. As with all quality improvement studies, the potential influence of the Hawthorne effect should be considered [30]. Increased awareness of being observed may have contributed to changes in procedural behaviour and documentation practices. All sites were metropolitan, public hospitals in a single high-income country which may limit generalisability to regional or lower-resource settings. More diverse, international data is needed to demonstrate the effect of synoptic reporting is universal. The study did not capture downstream treatment decisions or long-term outcomes, limiting assessment of clinical impact. Finally, the sample size was relatively modest, which may reduce precision in secondary analyses.

## 5. Conclusions

The implementation of synoptic reporting was associated with an increase in complete mediastinal staging across all five study sites. This study provides the first evidence supporting synoptic reporting as an effective quality improvement intervention in systematic staging EBUS.

More thorough staging translated into clinically meaningful outcomes, with increased lymph node sampling, greater sampling of radiologically normal nodes and a statistically significant increase in detection of PET-occult nodal metastases being observed, in addition to a reduction in variance in care. Although approaches to synoptic implementation varied between sites, the consistent improvement suggests the adoption of synoptic reporting is beneficial regardless of specific mode of delivery.

Future work should focus on wider implementation across diverse healthcare settings, optimisation of integration into clinical workflows, and evaluation of clinician acceptability and downstream impacts on patient outcomes.

## Figures and Tables

**Table 1:** Mandated synoptic reporting fields.

Item		Description
Indication (must include ‘Staging’)		Clinical reason for procedure Must explicitly include the term ‘staging’ to confirm that systematic nodal assessment was intended
Proceduralist name		The primary clinician performing the procedure
Clinical TNM stage prior to procedure		Clinical tumour (T), nodal (N), and metastatic (M) stage based on available clinical and radiological information.
For each of the 5 key mediastinal lymph node stations (2R, 2L, 4R, 4L, 7)		
	Was the node examined (yes/no)	Examination with endobronchial ultrasound
	Size of the node (in mm)	Short-axis diameter as measured by endobronchial ultrasound
	Was the node sampled (yes/no)	Whether tissue sampling was performed
	Number of needle passes	Samples taken from the individual lymph node
Use of rapid onsite evaluation (ROSE)		Use of immediate cytological assessment
Immediate adverse events		Complications occurring during and immediately after the procedure, whilst the patient was in the procedure room. This does not capture complications later in recovery or after discharge

**Table 2:** Baseline characteristics by study phase.

Characteristic	Pre (*n* = 96)	Post (*n* = 77)	Overall (*n* = 173)
**Age, years**			
Median (IQR)	70 (61–77)	70 (62–75)	70 (61–76)
**Gender, *n* (%)**			
Male	61 (63.5)	44 (57.1)	105 (60.7)
Female	35 (36.5)	33 (42.9)	68 (39.3)
**Pre-procedure diagnosis**			
Suspected NSCLC	95	73	168
Confirmed NSCLC	1	4	5

Percentages are calculated using total cases; no missing data were present. IQR: Interquartile range, NSCLC: Non-small cell lung carcinoma.

**Table 3:** Complete systematic mediastinal staging by site.

Site	Pre (*n*, %)	Post (*n*, %)	Odds Ratio (95% CI)	*p*-Value
1	1/32 (3.1%)	12/16 (75%)	78.0 (8.32–3967.3)	<0.0001
2	2/6 (33.3%)	8/10 (80%)	6.8 (0.54–137.1)	0.12
3	1/7 (14.3%)	8/9 (88.9%)	31.0 (1.75–2406.5)	0.009
4	3/29 (10.3%)	23/26 (88.5%)	57.4 (10.1–510.3)	<0.001
5	10/22 (45.5%)	16/16 (100%)	∞ (3.32–∞)	<0.001
Total	17/96 (17.7%)	67/77 (87%)	3.2 (13.6–74.4)	<0.0001

Percentages are calculated using total cases; no missing data were present. OR: Odds ratio; CI: confidence interval.

**Table 4:** Post-surgical upstaging.

	Pre-Intervention Cohort		Post-Intervention Cohort	
Site	Proceeded to Surgery	Upstaged at Surgery	Proceeded to Surgery	Upstaged at Surgery
1	12/32	0/12	8/16	0/8
2	0/6	0/0	1/10	0/1
3	0/7	0/0	3/9	0/3
4	6/29	1/6	10/26	1/10
5	5/22	1/5	6/16	0/6
Total	23/96	2/23	28/77	1/28

**Table 5:** Report completeness.

Reporting Domain	Pre (*n* = 96)	Post (*n* = 77)	*p*-Value *
Indication	18/96 (18.8%)	59/77 (76.6%)	<0.001
Name of proceduralist	86/96 (89.6%)	71/77 (92.2%)	0.607
Clinical stage (TNM)	4/96 (4.2%)	15/77 (19.5%)	0.002
Characteristics of lymph node (size in mm, morphology)	42/96 (43.8%)	75/77 (97.4%)	<0.001
Use of ROSE	67/96 (43.8%)	72/77 (97.4%)	<0.001
Immediate adverse events	69/96 (71.9%)	51/77 (66.2%)	0.507
All six mandated synoptic reporting fields completed	3/96 (3.1%)	13/77 (16.9%)	0.0027

Percentages are calculated using total cases; no missing data were present. * Fishers exact test; ROSE: Rapid Onsite Evaluation.

**Table 6:** Comparison of efficacy of synoptic reporting by integration into software.

Outcome	Non-Integrated Reporting Δ (Pre–Post)	Integrated Reporting Δ (Pre–Post)	Interaction *p*-Value *
Complete systematic mediastinal staging procedure	5/35 (14.3%) → 31/36 (86.1%)	12/61 (19.7%) → 36/40 (90%)	0.79
At least one radiologically normal node sampled	10/35 (28.6%) → 26/36 (72.2%)	27/61 (44.3%) → 29/41 (70.7%)	0.27
Mean number of nodes sampled	1.26 → 2.25 nodes	2.03 → 2.63	0.29
PET-occult lymph node detection rate	0/29 (0%) → 5/36 (13.9%)	3/61 (4.9%) → 4/41 (9.8%)	0.99

Percentages are calculated using total cases; no missing data were present. * Interaction *p*-value derived from linear mixed-effects models including an interaction term between study period (pre vs. post) and reporting type (integrated vs. non-integrated).

## Data Availability

Data is not publicly available. Further inquiries can be directed to the corresponding author.

## Supplementary Materials

The following supporting information can be downloaded at: https://www.mdpi.com/article/10.3390/cancers18101528/s1, Figure S1: All EBUS procedures performed during study period.

## Abbreviations

NSCLC	Non-small cell lung carcinoma
EBUS	Endobronchial Ultrasound
PET	Position Emission Tomography
CT	Computed Tomography
ROSE	Rapid Onsite Evaluation
IQR	Interquartile Range
