# Supplementary figures and images for: Synoptic Reporting Improves Quality of Endobronchial Ultrasound (EBUS): An Australian Multicentre Study
Source: Cancers (Basel). 2026 May 9;18(10):1528. doi: 10.3390/cancers18101528 (PMC13204654; doi:10.3390/cancers18101528)

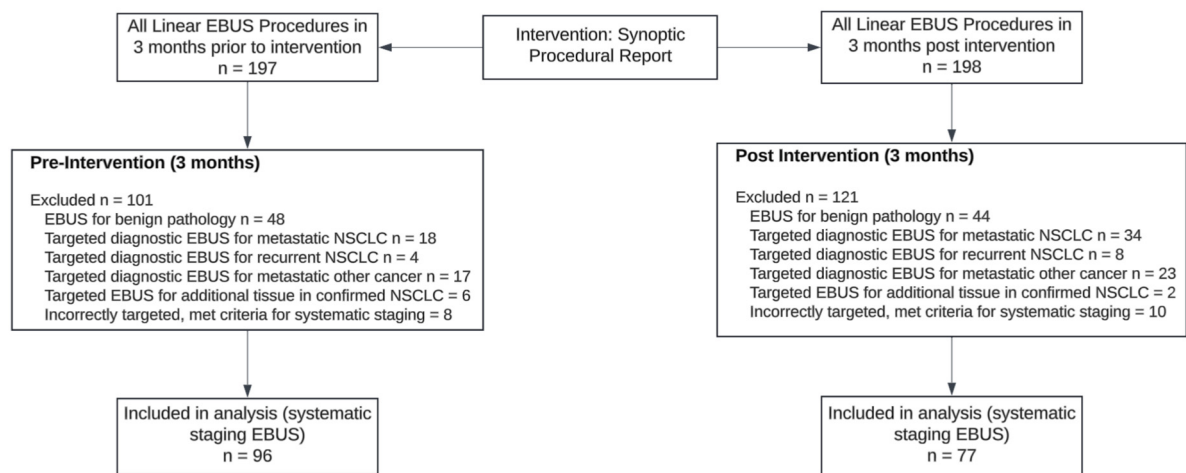

**Figure S1.** All EBUS procedures performed during study period.

Supplement: Supplementary file 1 [file cancers-18-01528-s001.zip › cancers-4261840-supplementary.pdf]
